# Dietary carbohydrate intake and the risk of esophageal cancer: a meta-analysis

**DOI:** 10.1042/BSR20192576

**Published:** 2020-02-25

**Authors:** Fei Xuan, Wei Li, Xiaoqing Guo, Chuanyong Liu

**Affiliations:** 1 The Second Department of Oncology, Jinan City People’s Hospital, Jinan, 271199, Shandong Province, China; 2 Department of Oncology, Jinan Central Hospital, Jinan, 250021, Shandong Province, China

**Keywords:** Carbohydrate, Dietary, Esophageal cancer, Meta-analysis

## Abstract

Background: Previous studies had been published to explore the association about carbohydrate intake on esophageal cancer risk, with inconsistent results. This meta-analysis aimed to assess the association between dietary carbohydrate intake and the risk of esophageal cancer.

Methods: Suitable studies were carefully searched with the databases of PubMed, Embase, the Cochrane Library, and Wanfang Database. A random-effects model was used for combined odds ratio (OR) and 95% confidence interval (CI). Stata software 14.0 was adopted for the analysis.

Results: At the end, 13 publications were included in our study. Pooled results suggested that highest category versus lowest category of carbohydrate intake could reduce the risk of esophageal cancer (summarized OR = 0.627, 95% CI = 0.505–0.778, *I*^2^ = 59.9%, *P*_for heterogeneity_ = 0.001). The results for carbohydrate intake on the risk of esophageal adenocarcinoma (summarized OR = 0.569, 95% CI = 0.417–0.777) and esophageal squamous cell carcinoma (summarized OR = 0.665, 95% CI = 0.453–0.975) were consistent with the overall result. A positive association was found in European, Asian, North American populations, instead of South American populations.

Conclusions: In conclusions, dietary carbohydrate intake may have a protective effect against the risk of esophageal cancer.

## Introduction

Cancer is a crucial health problem on a global scale that has become one of the primary causes of death. According to Globocan estimates in the year 2018, an estimated of 9.6 million were deaths from cancer [1]. Esophageal cancer remained an indispensable cause of cancer-related deaths and had shown a dramatic increase in global morbidity by more than six times [2]. Efforts to identify lifestyle factors [3] that may affect the risk of esophageal cancer had been ongoing, as well as some dietary factors, such as dietary vitamins [4,5], dietary fiber intake [6], dietary folate intake [7,8], total iron and zinc intake [9] and so on, may affect the development of esophageal cancer. Previous studies had been published to assess carbohydrate intake and some cancers risk, such as colorectal cancer [10], breast cancer [11], prostate cancer [12], but no meta-analysis was performed between carbohydrate intake and the risk of esophageal cancer. So far, numerous researchers explored dietary carbohydrate intake on the potential effects of esophageal cancer, but existing epidemiological data were inconsistent. Hence, we aimed to evaluate results from previous studies systematically and carefully by conducting a meta-analysis of observational studies to find: (1) whether highest versus lowest category of dietary carbohydrate intake could reduce the risk of esophageal cancer; (2) whether between-study heterogeneity or publication bias exited in our study.

## Method

We used the Meta-analysis of Observational Studies in Epidemiology (MOOSE) guidelines in the present study [13].

### Data source and search strategy

Two authors independently performed a literature search in databases of PubMed, Embase, the Cochrane Library, and Wanfang Database. All suitable studies published from beginning to July 1, 2019 were considered to be included. The associated medical subject headings and terms were ‘diet’ OR ‘dietary’ AND ‘carbohydrate’ OR ‘sugar’ AND ‘esophageal cancer’ OR ‘esophageal tumor’ OR ‘esophageal carcinoma’ OR ‘esophageal adenocarcinoma’ OR ‘esophageal squamous cell carcinoma’. Divergence in the search results was resolved by discussion.

### Inclusion criteria

The studies were included in our meta-analysis if they met the following criteria: (1) the studies were with case–control design or cohort design or cross-sectional study; (2) studies assessing the associations between dietary carbohydrate intake and the risk of esophageal cancer; (3) studies reporting in humans; (4) available odds ratio (OR) in case–control studies or relative risk (RR) in cohort studies and 95% confidence interval (CI) for highest category versus lowest category of dietary carbohydrate intake.

### Exclusion criteria

Overlapped studies or populations, conference reports, editor comments, reviews, case reports, and academic dissertations were excluded for the analysis.

### Data extraction

Two authors independently extracted the following data from each eligible study: first author’s name, publication year, research location, sample size, average cases age, disease type, study design, OR or RR and 95% CI of dietary carbohydrate intake, assessment of intake, adjusted or matched for factors. Divergence in the extraction was resolved by discussion.

### Quality assessment

The Newcastle–Ottawa Quality Assessment Scale was used to assess the quality of the included studies [14].

### Statistical analysis

Association analysis between dietary carbohydrate intake and the risk of esophageal cancer was performed using a random-effects model. The effect size was estimated by calculating the summarized OR or RR and its 95% CI [15]. The *I*^2^ statistic was used to estimate the degree of heterogeneity among the studies [16]. Meta-regression was performed to interpret the between-group heterogeneity [17]. Furthermore, sensitivity analyses and publication biases by Egger’s test [18] and Begg’s funnel plots [19] were performed. All tests were two-tailed, and a *P* value less than 0.05 were considered statistically significant.

## Results

### Characteristic of included studies

**Figure 1:**
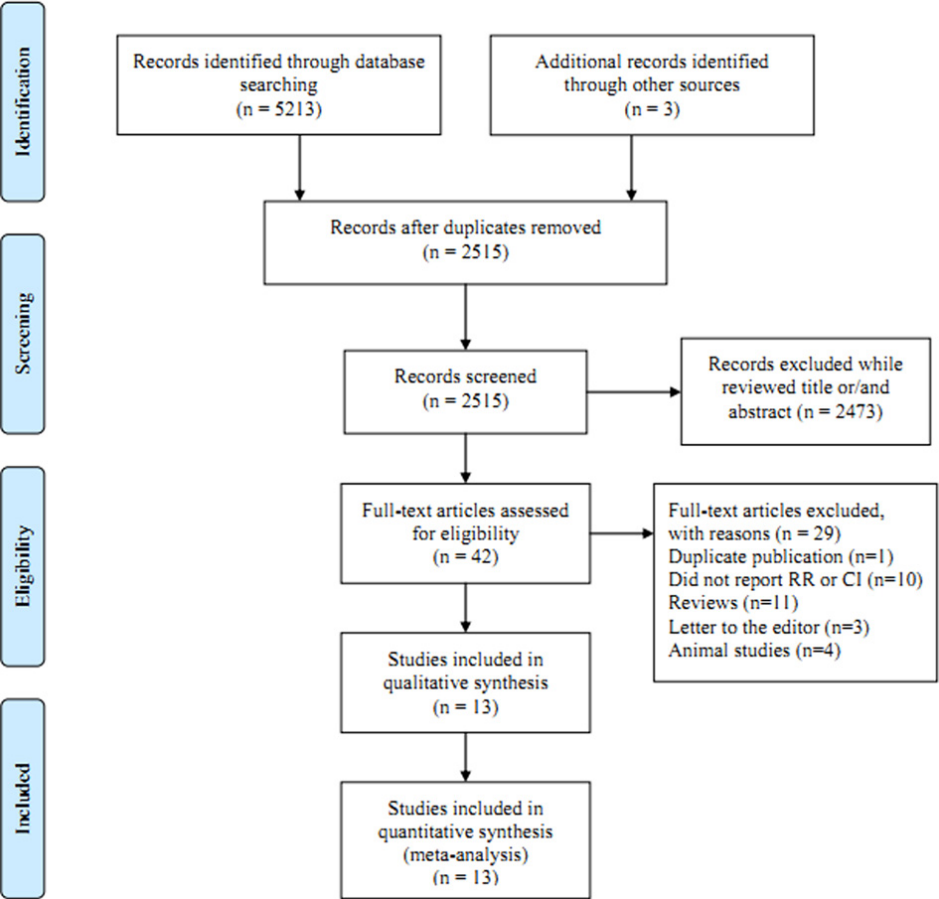
Flow chart of meta-analysis for exclusion/inclusion of studies

Our research returned 5213 articles from the above mentioned databases. Three articles were identified from the references of the relevant articles. After removing the duplicates from the different databases, 2515 articles were reviewed with titles and abstract. Then, 2473 articles were excluded due to not suitable for our analysis while reviewed the titles and/or abstract. The full texts of 42 articles were assessed. Twenty-nine articles were further excluded with reasons, which showed in the Figure 1. Finally, we included 13 articles [20–32] that assessed a total of 3033 patients in our meta-analysis. The quality evaluation scores (Table 1) of each study ranged from 6 to 9 and the methodological quality was higher. The characteristics of the included studies are shown in Table 1. Since all the included studies were with case–control design, we used the pooling OR instead of RR.

**Table 1:** Characteristics of the included studies

Study, year	Design	Age	Participants, Cases	Country	Disease type	Assessment of intake	Quality score	OR (95%CI) Highest versus lowest	Adjusted for or matched for
Chen et al., 2002	PBCC	62.3 ± 12.4	573, 124	United States	Esophageal adenocarcinoma	HHHQ	7	0.4(0.2–0.9)	Age, age squared, sex, respondent type, BMI, alcohol use, tobacco use, education, family history of cancers, and vitamin supplement use
De Stefani et al., 2006	HBCC	40–89	1170, 234	Uruguay	Esophageal squamous cell carcinoma	FFQ	8	0.74(0.47–1.17)	Age, sex, residence, urban/rural status, birthplace, education, body mass index, smoking status, years since quit smoking, number of cigarettes smoked per day, alcohol drinking, mate consumption, and total energy intake.
De Stefani et al., 1999	HBCC	NA	459, 66	Uruguay	Esophageal cancer	FFQ	6	0.8(0.5–1.1)	Age, sex, residence, urban/rural status, education, BMI, tobacco smoking, total alcohol intake, and total energy intake
Jessri et al., 2011	HBCC	40–75	143, 47	Iran	Esophageal squamous cell carcinoma	FFQ	8	0.22(0.05–0.84)	Age, sex, reflux, BMI, smoking, physical activity, and education
Lagergren et al., 2013	PBCC	<80	1008, 188	Sweden	Esophageal adenocarcinoma	FFQ	9	0.68(0.40–1.16)	Age, sex, reflux, BMI, smoking, alcohol consumption, education grade, and total energy intake
Lagergren et al., 2013	PBCC	<80	987, 167	Sweden	Esophageal squamous cell carcinoma	FFQ	9	1.05(0.61–1.80)	Age, sex, reflux, BMI, smoking, alcohol consumption, education grade, and total energy intake
Lahmann et al., 2014	PBCC	18–79	1778, 88	Australia	Esophageal adenocarcinoma	FFQ	8	0.79(0.49–1.25)	Age, sex, education, BMI, smoking, physical activity, alcohol intake, NSAID, diabetes, total fruit intake (except for fiber), red meat, processed meat, and total energy
Lahmann et al., 2014	PBCC	18–79	1717, 227	Australia	Esophageal squamous cell carcinoma	FFQ	8	0.46(0.28–0.75)	Age, sex, education, BMI, smoking, physical activity, alcohol intake, NSAID, diabetes, total fruit intake (except for fiber), red meat, processed meat, and total energy
Li et al., 2017	PBCC	30–79	2527, 500	United States	Esophageal adenocarcinoma	FFQ	8	0.93(0.56–1.54)	Age, sex, race, study indicator, BMI, fruits and vegetables intake, cigarette smoking, GERD frequency, and total energy intake
Mayne et al., 2001	PBCC	30–80	969, 282	United States	Esophageal adenocarcinoma	FFQ	7	0.34(0.20–0.58)	Age, site, sex, race, proxy status, BMI, income, education, smoking, and alcohol consumption
Mayne et al., 2001	PBCC	30–80	893, 206	United States	Esophageal squamous cell carcinoma	FFQ	7	0.68(0.37–1.25)	Age, site, sex, race, proxy status, BMI, income, education, smoking, and alcohol consumption
Mulholland et al., 2009	PBCC	64 ± 11	480, 224	Ireland	Esophageal adenocarcinoma	FFQ	8	0.39(0.16–0.98)	Age, sex, energy intake, smoking, BMI, education, occupation, alcohol, regular NSAID use, location, and H. pylori
Tzonou et al., 1996	HBCC	NA	256, 56	Greece	Esophageal adenocarcinoma	FFQ	6	0.84(0.59–1.19)	Age, sex, birth place, schooling, height, analgesics, coffee drinking, alcohol intake, tobacco smoking, and energy intake
Tzonou et al., 1996	HBCC	NA	243, 43	Greece	Esophageal squamous cell carcinoma	FFQ	6	1.12(0.75–1.69)	Age, sex, birth place, schooling, height, analgesics, coffee drinking, alcohol intake, tobacco smoking, and energy intake
Wolfgarten et al., 2001	PBCC	62.2 ± 1.9	140, 40	Germany	Esophageal adenocarcinoma	FFQ	8	0.07(0.03–0.40)	Age, gender, height, weight, BMI and socioeconomic data such as marital status and earning capacity
Wolfgarten et al., 2001	PBCC	58.1 ± 1.2	145, 45	Germany	Esophageal squamous cell carcinoma	FFQ	8	0.16(0.03–0.59)	Age, gender, height, weight, BMI and socioeconomic data such as marital status and earning capacity
Wu et al., 2007	PBCC	30–74	1514, 206	United States	Esophageal adenocarcinoma	FFQ	7	0.66(0.40–1.10)	Age, sex, race, birthplace, education, smoking, BMI, reflux, use of vitamins, total calories, and fat
Zhang et al., 1997	HBCC	NA	214, 90	United States	Esophageal adenocarcinoma	HHHQ	7	0.7(0.3–1.8)	Age, sex, race, education, smoking, alcohol intake, BMI, and total dietary intake in calories

Abbreviation: OR: odds ratio; CI: Confidence Intervals; PBCC: Population-based case–control study; HBCC: Hospital-based case–control study; NA: Not available; HHHQ: Health habits and history questionnaire; FFQ: Food frequency questionnaire; BMI: Body mass index.

In our included articles, there are five texts (Lagergren et al. 2013, Lahmann et al. 2014, Mayne et al. 2001, Tzonou et al. 1996, and Wolfgarten et al. 2001) reported both esophageal adenocarcinoma and esophageal squamous cell carcinoma about dietary carbohydrate intake. Therefore, 13 articles with 18 studies were used for the analysis.

### Main results

**Figure 2:**
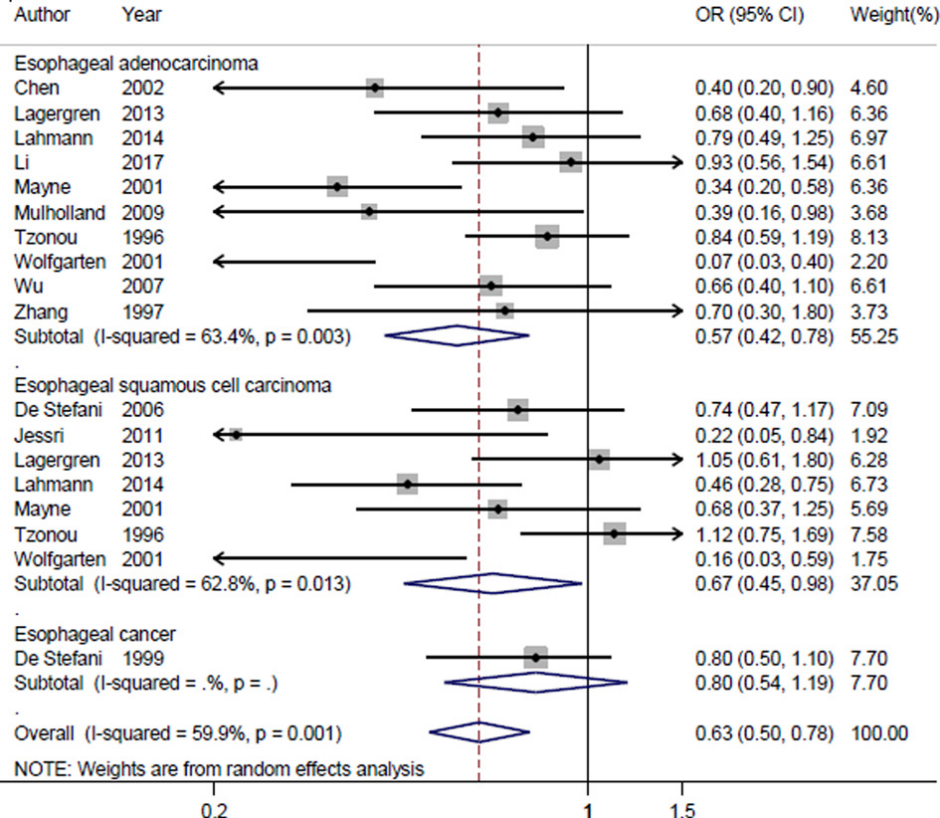
The forest plot of the association between dietary carbohydrate intake and esophageal cancer risk

In the results of the overall analysis, highest category versus lowest category of dietary carbohydrate intake could significantly reduce the risk of esophageal cancer (summarized OR = 0.627, 95% CI = 0.505–0.778, *I*^2^ = 59.9%, *P*_for heterogeneity_ = 0.001) (Figure 2). The results in the subgroup of esophageal adenocarcinoma (summarized OR = 0.569, 95% CI = 0.417–0.777) and esophageal squamous cell carcinoma (summarized OR = 0.665, 95% CI = 0.453–0.975) were consistent with the overall result. A positive association was found in European, Asian, North American populations, instead of South American populations. When we conducted a subgroup analysis by study design, the association was significant in population-based case–control studies (PBCC), but not in the hospital-based case–control studies (HBCC). The detailed results are shown in Table 2.

**Table 2:** Summary results about the association between dietary carbohydrate intake and esophageal cancer risk

Subgroups	Number of studies	Number of cases	OR(95% CI)	P for trend	Heterogeneity test	
					*I*^2^ (%)	*P*
Total	18	3033	0.627(0.505–0.778)	<0.001	59.9	0.001
Disease type						
Esophageal adenocarcinoma	10	1998	0.569(0.417–0.777)	<0.001	63.4	0.003
Esophageal squamous cell carcinoma	7	969	0.665(0.453–0.975)	0.037	62.8	0.013
Study design						
PBCC	12	2497	0.541(0.401–0.729)	<0.001	63.2	0.002
HBCC	6	536	0.831(0.669–1.030)	0.091	15.3	0.316
Geographic locations						
Europe	7	763	0.586(0.364–0.943)	0.028	75.5	<0.001
Asia	3	562	0.534(0.308–0.927)	0.026	53.9	0.114
North America	6	1408	0.590(0.425–0.820)	0.002	43.1	0.118
South America	2	300	0.774(0.574–1.043)	0.092	0.0	0.800

OR: odds ratio; CI: confidence interval; PBCC: population-based case–control studies; HBCC: hospital-based case–control studies

### Publication bias and sensitivity analysis

**Figure 3:**
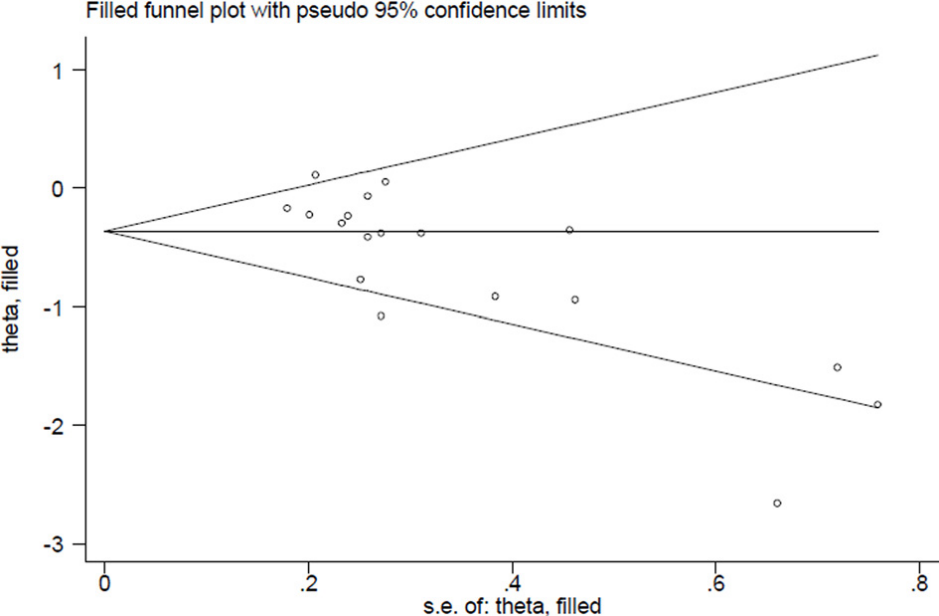
Funnel plot for the analysis of publication bias between dietary carbohydrate intake and esophageal cancer risk

**Figure 4:**
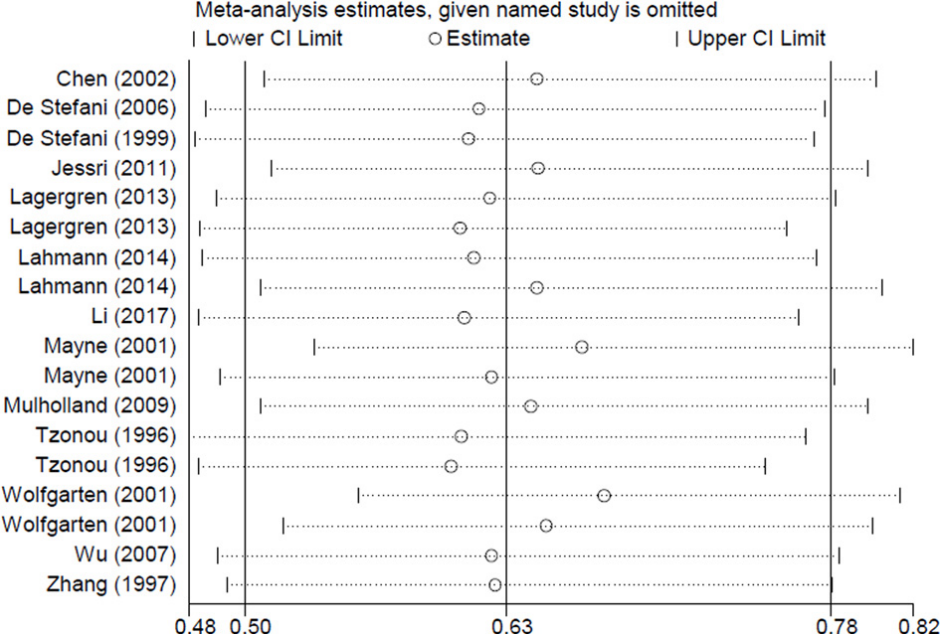
Sensitivity analyses between dietary carbohydrate intake and esophageal cancer risk

In the publication bias assessment, the results from funnel plots (Figure 3) and Egger’s test (*P* = 0.107) detected no publication bias. Sensitivity analyses (Figure 4) showed no single study had essential effect on the overall result.

## Discussion

Numerous of studies about dietary carbohydrate intake and esophageal cancer had been published, with conflicting results. However, no meta-analysis was performed to obtain a definitive conclusion. Therefore, we conducted this study to clarify whether dietary carbohydrate intake had some inverse effects on the development of esophageal cancer. In total, our results suggested that dietary carbohydrate intake had significant association on the lower development of esophageal cancer.

In the current meta-analysis, people with higher carbohydrate intake may reduce the risk of esophageal cancer. On the one hand, carbohydrate intake was negatively correlated with fat intakes. Therefore, people who were with higher carbohydrate intake may also have lower intake of fat, then explained its inverse association with esophageal cancer [23]. On the other hand, people who were with higher intake of carbohydrate could be reflection of more plant-based food intakes, and especially fruit and vegetable, which had been confirmed having a relationship with esophageal cancer [33].

We found significant between-study heterogeneity in the whole pooled results of dietary carbohydrate intake and esophageal cancer risk. As introduced in the methods, we used meta-regression to explore the causes of heterogeneity for covariates of publication year, disease type, study design, geographic locations, assessment of intake and number of cases. Results from meta-regression suggested that no covariates increased the high between-study heterogeneity. Moreover, between-study heterogeneity also exited in the subgroup analyses by disease type, study design and geographic locations. We then used leave-one-out analysis to reduce the between-study heterogeneity. The *I^2^* was reduced to 47.1% when we leaved one study by Wolfgarten et al. 2001 [30] (about the esophageal adenocarcinoma study). And the pooled result about the remaining 17 studies was not changed (summarized OR = 0.673, 95% CI = 0.558–0.811). Meanwhile, all the pooling results in our analysis are based on adjusted OR in each individual study, and thereby could control some between-study heterogeneity.

The present study still had several limitations. First, all the included studies were case–control studies. As well as known, the selection bias, recall bias and some other confounding factors cannot be excluded in the case–control studies. Hence, it is requirement for evidence from prospective cohort studies. Second, all the included studies were with English language and this may omit other languages studies. Meanwhile, the papers which had been published in the journal or online were searched and included in our analysis. Those papers which published in the meetings or unpublished were not searched. However, we did not detect any publication bias in our meta-analysis. Third, we only assessed the association between dietary total carbohydrate intake and the risk of esophageal cancer, and did not assess the association between carbohydrate type and esophageal cancer risk due to the limitation data provided in all the included original articles. Hence, more articles with detailed carbohydrate type are warranted to further assess the risk of esophageal cancer.

## Conclusions

In summary, our results indicated that dietary intake of carbohydrate may contribute to the lower development of esophageal cancer. As some limitations existed in our analysis, large scale prospective studies with detailed type of dietary carbohydrate intake are needed to verify our results.

## Abbreviations

CI: confidence interval
HBCC: hospital-based case–control studies
MOOSE: Meta-analysis of Observational Studies in Epidemiology
OR: odds ratio
PBCC: population-based case–control studies
